# The experience of memory: it's unconscious origins

**DOI:** 10.3389/fcogn.2024.1358396

**Published:** 2024-01-30

**Authors:** Denis Brouillet

**Affiliations:** ^1^Epsylon Laboratory, Université Paul Valéry Montpellier, Montpellier, France; ^2^LICAE Laboratory, Université Paris Nanterre, Nanterre, France

**Keywords:** memory, fluency, active inference, consciousness, pastness

## 1 Introduction

In this article, we propose to explore the non-conscious component of the experience of memory. More specifically, on the basis of both empirical data concerning the role of the presence of a discrepancy in ongoing processing and on theoretical arguments from the framework of active inference, we propose a way of understanding how the experience of memory - the phenomenal sensation of the past - emerges.

## 2 A gap in the expected fluency underlies feeling of pastness

Since the work of Jacoby and Dallas (1981), several authors consider that the experience of memory originates from an inference based on a phenomenological cue, *fluency*. This refers to the subjective (metacognitive or qualitative) experience associated with all cognitive processing. However, as fluency is phenomenologically transparent to its own causal source (Metzinger, 2003), in the sense that it does not represent such a source - it is just a subjective feeling - identifying the source relies on an attribution process based on inference: if fluency is experienced, its origin can only be what is in the attentional focus, the processed stimulus.

However, the subjective feeling of fluency can arise because the ease with which the event is processed becomes surprisingly easily than expected (Whittlesea and Leboe, 2003). Indeed, faced with an event, people have a preconceived subjective idea of how it will be processed (easily vs. with difficulty). If the a priori feeling is that its processing will not be easy, people will be surprise if the ongoing processing is easier than expected. Consequently, this sense of surprise leads peoples to attribute to the event its origin and they infer that if it's easier than they thought, it means this event is not new to them.

However, a question remains: is the cognitive system sensitive to the existence of a discrepancy only when supported by the processed stimulus, or is it sensitive to the existence of a discrepancy itself? Some studies have answered this question. For example, Brouillet et al. (2017) observed that a fluent gesture, unrelated to the semantics of the words to be recognized, performed before their appearance, improves their recognition. This effect is particularly pronounced for new words (46% recognized), which is particularly revealing because, unlike old words for which repetition leads to an increase in fluency, the discrepancy is most surprising for them, as there is no sense of fluency. In a more recent article, Brouillet et al. (2022a,b) showed that a transfer of the feeling of motor fluency, associated with a task performed before the memory task and unrelated to it, affected participants' performance. Again, for new non-words this effect is particularly significant (35% recognized).^1^ In short, these two studies show that the discrepancy in itself can have an impact on memory performance. These results are in line with work in neuroscience which has shown that the mismatch between expected and current events is at the origin of memory reconsolidation (i.e., the updating of memories), even if it does not take place consciously (Fernández et al., 2016). So, it seems that the cognitive system is sensitive to the perception of a discrepancy (surprise) between its expectations.

It is certainly the theoretical framework of Active Inference (for a complete overview: Parr et al., 2022) that seems to offer the most relevant way of explaining this. Indeed, it assumes that surprise plays a key role in cognitive processing because it is an attribute of sensations.

## 3 Active inference a theoretical framework to understand the discrepancy effect

Active inference uses the principle of Bayesian inference applied to the brain: it infers a probabilistic generative internal model of the world from sensory inputs. In turn, it makes it possible to anticipate likely sensory inputs and to assess the difference between these and those predicted by the internal model. In other words, the brain is constantly making predictions about sensory inputs in order to generate an error or surprise signal that will allow the necessary adjustments to be made.

While the brain's main function is minimizing prediction errors, what is more important is to minimize only those that convey relevant information to updating perception or action. But what matters therefore, is the accuracy of predictions. In order to assess the accuracy of its predictions, our brain must have beliefs (confidence) about the accuracy of its own predictions. Brouillet and Friston (2023) have argued that fluency plays a central role in prediction because it reflects or enables the recognition of the accuracy of predictions and thus underlines the accurate updating of beliefs. However, according to the authors, it is not the fluency itself that is perceived first, but rather the unconscious recognition of a change in the attentional system: our attention is drawn to something that we have not predicted. These unconscious changes can be perceived as non-felt fluency - *unfelt fluency*. In this view, unfelt fluency is at the basis for evaluating of the accuracy of our predictions and the feeling of fluency is an attributional inference about the optimization of the resulting active inference. In other words, felt fluency can be interpreted as the “awareness” of non-felt fluency. Indeed, although felt fluency is phenomenologically transparent (see above), it renders unfelt fluency opaque. But it is unfelt fluency that is important for active sensing because it functions as a non-conscious process that instantiates attention in response to surprise (unresolved prediction errors) and triggers an active inferential process that results in a selection of actions that modify the incoming sensory data, source of felt fluency.

## 4 The non-conscious component of the memory experience

We believe that this explanation is a good way of understanding the results on the items New of the experiments that highlighted the discrepancy effect on memory performance, regardless of the words to be remembered. Indeed, before attributing felt fluency to the words, we must consider that an active inferential process takes place to give meaning to what is felt. In order to understand this, we have to bear in mind that these experiments concern a recognition task. So, when a word appears, people are in a state of uncertainty, leading them to believe that the task will not be easy and that drives them to search for cues that help reduce this uncertainty. Among the relevant cues indicating that a word has been seen, fluency plays a central role (see above). Therefore, when an old word appears, its repetition itself carries fluency, and the participant can rely on it to respond. However, when a new word appears, it doesn't generate fluency by itself, yet within the task context, a sense of fluency is experienced. Thus, a discrepancy is felt between what is expected (i.e., non-fluency) and what is experienced (i.e., fluency). Here, the contribution of active inference involving fluency helps to provide a possible explanation for what is happening and helps to understand why new words are being recognized to a relative extent.

Non-fluency felt - unfelt fluency - associated to the new words triggers attention to the ongoing process in response to uncertainty and initiates an active inferential process ('If I sense a discrepancy, then there is a cause to it') finding the cue or cues causing the discrepancy. This process of active inference involves directing attention to aspects of the sensorium that were not the focus of attention. For example, ease of word reading will lead to a sensation of fluency (see for example Whittlesea and Williams, 1998). As fluency serves as a reliable cue from the past, the active inferential process results in a judgement of recognition. Therefore, the recognition judgment is not the result of a direct attribution to the event; it is mediated by an active inference that originates from a perceived unfelt fluency and manifests in a felt fluency. This also helps understand why old words are better recognized than new ones, they combine both sources of fluency: the one linked to repetition and the one associated with active inference.

## 5 Conclusion

The purpose of this article was to contribute to understanding what constitutes a memory experience, specifically the awareness that an event is related to the past. The central idea put forward is that a memory experience is consistently preceded by a phenomenal experience rooted in a subjective evaluation of the accuracy of our predictions (see Deane, 2021). This evaluation results from the implementation of an inferential process based on the subjective perception of a discrepancy between what is expected (e.g., uncertainty about whether the word was previously seen) and what is perceived (e.g., a vague sense that this word is not surprising).

Based on experimental data and the Active Inference framework, we propose that conscious memory inference, which involves attributing the perceived fluency to the event being judged - a guarantee that this event is not new - is preceded by unconscious perception that serves as both cause and consequence. This unconscious sensing, referred to as unfelt fluency, inherent in the presence of a discrepancy, reveals a change in the attentional field related to the stimulus. It acts as a cause because it originates the redeployment of attention and as a consequence by guiding attentional selection. In other words, the unfelt fluency is the recognition that our attention is captured by something we cannot explain – or have not predicted - leading to an emphasis on current sensory input stemming from the stimulus being processed. This manifests as felt fluency - recognizing of fluency- that arouses awareness of the past: an experience of memory emerges.

In consciousness theories, such a view is part of a functional approach to consciousness where attention plays a crucial role in altering the content of consciousness (for a critical review of theories of consciousness, read Seth and Bayne, 2022). But for the deployment of attention to be successful, it is necessary for the system to make top-down predictions about expected states in order to predict error accuracy. In other words, the system needs to predict its own states in order to deploy attention. As Dołega and Dewhurst (2021) suggest, these top-down meta-predictions can be compared to Dennett (2005) probe. The consequence is that there are no facts about what a system is aware of until there has been a probe. We believe that unfelt fluency acts as probing.

In short, prior to being a consciousness of pastness, the memory experience is a kind of “resonance” emerging from an initially non-recognized fluency overshadowing by recognized fluency. It is this phenomenal resonance that creates an interpretative context from which awareness originates and, in a recognition task, the experience of memory. In essence, experience of memory, consciousness of pastness, is no more than an active inference originates from unfelt fluency.

## Author contributions

DB: Writing—original draft.

## Footnotes

^1^In the original experiment on the discrepancy hypothesis (Whittlesea and Williams, 1998), the rate of recognition of New was 35%.
